# Extracellular Vesicle-Dependent Cross-Talk in Cancer—Focus on Pancreatic Cancer

**DOI:** 10.3389/fonc.2020.01456

**Published:** 2020-08-19

**Authors:** Lise Nannan, Jean-Baptiste Oudart, Jean Claude Monboisse, Laurent Ramont, Sylvie Brassart-Pasco, Bertrand Brassart

**Affiliations:** ^1^Université de Reims Champagne Ardenne, SFR CAP-Santé (FED 4231), Laboratoire de Biochimie Médicale et Biologie Moléculaire, Reims, France; ^2^CNRS UMR 7369, Matrice Extracellulaire et Dynamique Cellulaire–MEDyC, Reims, France; ^3^Biomedical MRI Group, Department of Imaging and Pathology, KU Leuven, Leuven, Belgium; ^4^CHU Reims, Service de Biochimie–Pharmacologie–Toxicologie, Reims, France

**Keywords:** extracellular vesicle (EV), pancreatic cancer, biomarkers, imaging *in vivo*, bioactivities

## Abstract

Extracellular vesicles (EVs) like exosomes and shed microvesicles are generated by many different cells. However, among all the cells, cancer cells are now recognized to secrete more EVs than healthy cells. Tumor-derived EVs can be isolated from biofluids such as blood, urine, ascitic fluid, and saliva. Their numerous components (nucleic acids, proteins, and lipids) possess many pleiotropic functions involved in cancer progression. The tumor-derived EVs generated under the influence of tumor microenvironment play distant roles and promote cellular communication by directly interacting with different cells. Moreover, they modulate extracellular matrix remodeling and tumor progression. Tumor-derived EVs are involved in pre-metastatic niche formation, dependent on the EV-associated protein receptors, and in cancer chemoresistance as they transfer drug-resistance-related genes to recipient cells. Recent advances in preclinical and clinical fields suggest their potential use as biomarkers for diagnosis and prognosis as well as for drug delivery in cancer. In this Review, we discuss EV characteristics and pro-tumor capacities, and highlight the future crucial impact of tumor-derived EVs in pancreatic cancer diagnosis and prognosis.

## Introduction

Intercellular communication is essential to cell development and maintenance of homeostasis in multicellular organisms. Naturally produced and released into the extracellular microenvironment by most cell types and belonging multiple distinct classes depending on their origin, EVs are predominantly described in intercellular communication although their functions are not limited to this aspect (1). These cell-to-cell communications occur locally or at distance. Distant intercellular communication is achieved via EVs. Among all EVs, two major EV classes are relatively well-described: exosomes (50–150-nm diameter, membranous vesicles of endocytic origin) and microvesicles (large membranous vesicles of 100–1,000-nm diameter directly shed from the plasma membrane) (2). Since the 1990's with the first evidence on EV roles in cell-to-cell communication and more especially during the past 10 years, considerable progress have been made to understand EV functions (3–5) and potential applications in clinical domains (6–9). Numerous studies have shown the biological roles of EVs in physio-pathological processes, such as immune and microbiological regulation, stem cell biology, cardiovascular diseases, neurodegenerative diseases, metabolic disorders, and cancer progression (10–13). The regulation of a broad range of cellular activities and biological responses is due to their biogenesis and probably to the extracellular environment and constraints. EVs are specifically loaded with cell-specific proteins, lipids, mRNAs, and miRNAs, corresponding to EV membrane's or cargo's “molecular signature,” which reflect activity/status of the parent cancer cells (2). Kahlert et al. demonstrated that pancreatic cancer cell-derived EVs contain fragments of genomic DNA (14). The deregulation of EV biogenesis in different pathologies, especially cancer, was also evidenced furthering tumor cell immune escape, therapy resistance, tumor growing, invasion, and metastasis (15).

Practically, most studies are focused on EVs that measure 1 μm or less, corresponding mostly to exosomes. To date, few articles are based on microvesicles larger than 1 μm like large oncosome (16–19). In 2014, The International Society for Extracellular Vesicles (ISEV) proposed Minimal Information for Studies of Extracellular Vesicles (“MISEV”) guidelines for EV isolation and purification. Among the different EV isolation methods, such as membrane filtration, affinity isolation, or size exclusion chromatography from conditioned cell culture media and body fluids (plasma, serum, and urine), the must employed method is the ultracentrifugation (20). According to the MISEV2014, EV detection and characterization must be realized by transmission electron microscopy and EV marker immunodetection by western-blotting or flow cytometry. Specific exosome markers correspond to tetraspanins (CD9, CD63, and CD81), ESCRT-associated proteins like tumor susceptibility gene 101 (TSG101) and apoptosis-linked gene 2-interacting protein X (ALIX), heat shock proteins (Hsp70 and Hsp90), integrins, and membrane transport and fusion proteins (annexins). To date, microvesicle characterization is less obvious due to the absence of specific markers. Recently, following the increase in studies focusing on EVs, many critics have emerged. EV nomenclature, collection, pre-processing, separation, and concentration methods, quantification and characterization are now required. The MISEV2018 guideline harmonizes these aspects and avoids misunderstandings such as the presence of potential contaminant in EV preparations (21).

## Biogenesis of EVs

Classification in microvesicle or exosome depends on EV biogenesis mode. However, depending on the cell source, exosome, and microvesicle biogenesis can share similarities. Exosome and microvesicle biogenesis share common molecular components and biogenesis mechanisms at the plasma membrane or at the endosomal membrane (1). EV biogenesis mechanisms have only recently started to be uncovered. Tumor microenvironmental modifications appear to play a crucial role in EV release. Ionic homeostasis changes were reported to influence EV biogenesis. Intracellular Ca^2+^ increase induces the collapse of plasma membrane phospholipid asymmetry, the destabilization of plasma membrane-cytoskeletal anchorage and finally the release of EVs (18–22). Microenvironmental acidosis promotes tumor progression by stimulating invasion and metastasis and was reported to stimulate EV shedding (23–25). Extracellular matrix (ECM) degradation and bioactive peptide release, such as elastin-derived peptides, increase EV release by cancer cells (18). To date, independent pathways have been shown to be involved in EV biogenesis: the endosomal sorting complex required for the transport (ESCRT) dependent pathway, the asymmetry lipid involvement required for the budding formation and release, the tetraspanin-dependent pathway responsible for selecting cargoes for exosomes, the syndecan and syntenin pathways required for budding (2, 26–28). The intracellular trafficking involved in exosome and EV secretion is mediated by Rab GTPase proteins (Rab proteins and SNARE proteins) that control intracellular vesicle trafficking, exosome release and the fusion of lipid bilayer at the plasma membrane. The cytoskeletal components play a crucial role in the budding and the release of EVs (29). Actomyosin cytoskeleton reorganization is necessary to microvesicle formation and probably to exosome release at the plasma membrane.

## Bioactivities of Pancreatic Ductal Adenocarcinoma (PDAC) EVs

Like in many cancer, the PDAC microenvironment is complex. It results from communications between PDAC cells, stromal cells such as fibroblasts, myofibroblasts, stellate cells, vascular endothelial cells, immune cells, all embedded in an abundant ECM (30). Cancer-associated fibroblasts (CAFs) promote ECM remodeling and tumor growth. Immune cells have a highly immunosuppressive activity and further contribute to immune evasion. Interactions between cellular and acellular components of the PDAC-tumor microenvironment promote tumor progression, contribute to metabolism alterations, cancer cell proliferation, tumor metastasis, and abnormal tumor-associated immunity (31). In this environment, EVs play a crucial role (32–34). Stromal EVs promote invasive behavior and upregulate drug resistance and immune escape pathways in cancer cells. PDAC EVs induce stromal cell phenotype changes, from fibroblasts to CAFs for example, promote tumor cell proliferation, invasion, and metastasis; they also modulate tumor-associated immunity (35). Pancreatic exosomes were also reported to induce cell death and to inhibit Notch-1 pathway (36). Microvesicules derived from human pancreas carcinoma cells were reported to induce IL-10 synthesis in human classical monocytes *via* hyaluronan, which in turn activates the PI3K/Akt/mTOR pathway (37).

Whatever the nature of the EV/cell interaction, EVs play cargo molecule function, and protect their contents from degradative enzymes like RNases and proteinases due to their double lipid membrane (1). After their release in the extracellular microenvironment, EVs target recipient cells and deliver their content that induce functional responses and modulate phenotypic changes with physiological and pathological consequences. This EV-dependent cell-to-cell communication requires receptor-related events like docking at the cell membrane, activation of cell surface receptors, and intracellular signaling, vesicle endocytosis, or membrane fusion with target cell (1). These aspects of the EV-derived intercellular communication are not fully understood. Due to different factors (EV origin and type, identity and origin of the targeting cells), these processes are complex and determine downstream effects and processes. Current studies mainly focused on membrane interaction, receptor/ligand identification, and intercellular fate of EV pools. The mechanisms of EV uptake and cargo delivery into the target cells are still incompletely characterized. Depending on the EV origin and the target cell type, this step may be either very specific (ligand-receptor interactions: integrins/ICAM interaction, lectin/proteoglycan interaction, lipid-binding protein/phosphatidylserine interaction) or very general (micropinocytosis, phagocytosis, caveolae-, or clathrin-dependent mechanisms, membrane fusion) (1, 2). EVs may transfer informations to target cells by acting at the cell surface as cargo of ligand to cell membrane receptor, without delivery of their content. The presence of a specific protein on EV surface can lead to positive- or negative-election mechanisms. The CD47 integrin-associated protein is often present at the EV surface and increases the time of EV circulation in the blood by preventing their phagocytosis by macrophages and monocytes (38). This results in an increased EV uptake by pancreatic cells. All these biological activities, associated with the increased production of EV in PDAC, are consistent with a potential role of EVs as biomarkers of PDAC.

## EVs as Biomarkers for Diagnosis and Prognosis in Pancreatic Cancer

EVs, especially exosomes, are considered a future potent tool for both diagnostic, prognostic, and therapeutic applications, being a natural way for efficient biodelivery. EVs of defined cell types may serve as novel tools for various therapeutic approaches, including anti-tumor therapy, pathogen vaccination, immune-modulatory, and regenerative therapies or drug delivery (6). The emerging field of basic and applied EV research will significantly influence the biomedicinal landscape in the future. EVs contain specific molecules of originate cells, display stability, and abundance in various biofluids, that may largely increase sensitivity and specificity in PDAC diagnosis. Many clinical trials show increased number of biofluid exosomes in cancer patients compared to healthy people (39–42), suggesting that the measure of the levels of circulating exosomes could represent a disease marker *per se* (43). This may be the case of pancreatic cancer as well. In this section, we will focus on the impact of the EVs on the diagnosis and prognosis of PDAC. The continuing increase in PDAC incidence leads it to be the second leading cause of cancer mortality in 2030 (44). The dismal prognosis of PDAC is mainly attributed to poor detection rates at early stages, rapid progression, and disappointing surgical resection outcomes. Most patients with PDAC lack diagnostic symptoms during early stages, and existing early screening biomarker lack (35). Diagnosis mainly relies on medical imaging and pathological confirmation on tissue sample analysis (45). The identification of blood markers remains an important challenge in daily practice, since the tumor marker CA19-9 showed a lack of sensitivity and specificity in non-advanced PDAC. To increase pathological diagnosis performance, different biomarkers have been studied in order to differentiate PDAC from benign lesions. Molecular markers such as exosomal DNA mutation or exosomal miR expression and exosomal surface biomarkers such as integrins or glypican1 (GPC1) remains the most promising exosomal biomarkers of PDAC.

### EV Proteins as Potential Biomarkers of PDAC

Differences in composition between exosomes from human non-malignant epithelial and pancreatic cancer cells were analyzed by Emmanouilidi et al. (46). Proteomic analysis reveals the selective enrichment of known exosome markers and also signaling proteins involved in pancreatic cancer progression (KRAS, CD44, and EGFR) in oncogenic exosomes compared to exosomes from non-malignant cells. Moreover, oncogenic exosomes contain factors known to regulate the pre-metastatic niche (S100A4, F3, ITGβ5, and ANXA1), clinically-relevant proteins which correlate with poor prognosis (CLDN1, MUC1) as well as protein networks involved in various cancer hallmarks including proliferation (CLU, CAV1), invasion (PODXL, ITGA3), metastasis (LAMP1, ST14) and immune surveillance escape (B2M). This study highlights the modification of exosome protein content during tumorigenesis and suggests putative components as prognostic and diagnostic biomarkers in pancreatic cancer (46).

To date, only described in prostate cancer, the microenvironmental pressure induces a preferential expression of known tumor markers like Prostate Specific Antigen (PSA) on the released exosomes. Increased exosomal PSA expression has been shown to represent a valuable biomarker for both screening and secondary prevention of prostate cancer in clinical studies (47). Up to now, PDAC exosome researches do not provide such a promising tumor-specific biomarker.

However, different cell surface biomarkers were assessed for the diagnosis or prognosis of PDAC. Among these potential biomarkers, integrins and GPC1 are of particular interest. The implication of integrins in the determination of organotropic metastasis was investigated by Hoshino et al. in 2015 (48). They showed that the capture of tumor-derived exosomes by organ-specific cells prepares the pre-metastatic niche. Proteomic analysis revealed that tumor-derived exosomes harbored specific integrin patterns associated with the organ-specific metastases. Exosomal α_6_β_4_ and α_6_β_1_ integrins were associated with lung metastasis, whereas α_v_β_5_ integrin was related to liver metastasis. Moreover, exosomal integrin patterns could also modulate the interaction between exosomes and ECM components in specific organs. Interestingly, α_v_β_5_ integrin mainly expressed in BxPC-3-LiT pancreatic cell line derived exosomes co-localized with liver macrophages in fibronectin-rich microenvironments. Accumulating evidence showed that exosomes are not only protein or nucleic acid cargos. Recent proteomic studies showed that the amount of α_6_, α_v_, and β_1_ integrin-subunits in tumor-derived exosomes was correlated with tumor stages in different epithelial cancer cells and could be considered as a putative circulating biomarker of some primary tumors (49). As well, the detection of exosomal α_v_β_3_ integrin in prostate cancer patients could be a clinically useful biomarker of prostate cancer progression (50).

GPC1 is a cell surface heparan sulfate proteoglycan, barely expressed in normal pancreatic tissue. Its transcript is silenced in non-tumoral tissue whereas it is re-expressed in PDAC due to potential epigenetic variation of promotor methylation (51). Immunochemistry analysis of PDAC tissues showed that highly positive expression is associated with shorter overall survival (OS) and could be considered as a diagnostic and prognostic marker of PDAC (52). On the other hand ELISA based assay of serum GPC1 do not allow to distinguish PDAC patients from controls and cannot be used as a diagnostic biomarker of PDAC patients. Nevertheless, GPC1 appeared specifically enriched in circulating pancreatic cancer cell-derived exosomes (53, 54). Using flow cytometry analysis of serum, Melo et al. reported that GPC1 positive exosomes allowed to distinct healthy subjects or benign pancreatic disease patients from pancreatic cancer patients. Moreover, exosomal GPC1 level correlates with tumor burden and patient survival (53, 54). GPC1 was also suggested as an early diagnostic and prognostic marker as well as a therapeutic target for PDAC by Haizhen et al. (51). By contrast, a recent study showed that GPC1 positive EVs do not allow to distinct PDAC from benign pancreatic diseases (55). Despite these conflicting results, exosomal GPC1 remains a potential biomarker of interest in the diagnosis or prognosis of PDAC but needs further investigations to be validated in daily practice.

Cytoskeleton-associated protein 4 (CKAP4), a novel Dickkopf WNT Signaling Pathway Inhibitor 1 (DKK1) receptor was highly detected in the serum of PDAC patients, whereas it was barely detectable in serum from postoperative patients. Exosomal CKAP4 may represent a PDAC biomarker and anti-CKAP4 mAbs can contribute to the development of novel diagnostic methods and therapeutics (56).

Large extracellular vesicles, specifically AnnexinV^+^ EpCAM^+^ CD147^+^ tumor-associated microparticles were reported to facilitate the detection of pancreas carcinoma (57).

Venous thrombo-embolic event (VTE) biomarkers including D-dimers and microvesicle-tissue factor (MV-TF) were reported to be related to cancer process and dissemination. D-dimers and MV-TF activity are associated to future VTE in PDAC patients and could help to identify patients who could benefit from thromboprophylaxis (58).

### *KRAS* Mutation in Circulating EVs as a Biomarker of PDAC

Up to 80% of PDAC cells harbored *KRAS* mutations. *KRAS* analyses can be performed either on tissue or plasma samples. However, circulating tumor DNA (ctDNA) can only be detected in 30–68% of resectable tumors and in 70–80% of advanced PDAC. This may be explained by a limited amount of ctDNA released by tumor cells at early stages of the disease or by the degradation of ctDNA by DNases (59). Nevertheless, detection of ctDNA seems to be correlated with the prognosis of PDAC. For example, Lin et al. showed that ctDNA was detected in 29.2% of PDAC patients and its detection of ctDNA was associated with a significantly shorter overall survival (OS) (60). Kalluri et al. showed that exosomes contained double stranded DNA (61). Exosomal DNA seems to be protected from blood DNAse degradation which potentially allows the identification of a higher rate of mutations. As reported in plasma samples, circulating exosomal DNA analyses allow the detection of *KRAS* mutation in PDAC. Yang et al. reported 39.6% of *KRAS* c.35G>A (p.Gly12Asp) mutation in 48 PDAC derived exosomal plasma samples. Moreover, *KRAS* mutations have also been isolated in non-tumoral samples (i.e., chronic pancreatitis) and more surprisingly in three of 114 (2.6%) healthy subject samples (62). *KRAS* mutations in presumed healthy subject were also identified in different studies both in ctDNA and exosomal DNA (26, 63). Moreover, *KRAS* mutation allele frequency (MAF) from exosomal DNA is significantly associated with disease progression after neoadjuvant chemotherapy in a prospective cohort of potentially resectable pancreatic tumor. In addition, exosomal *KRAS* MAF>5% is associated with shorter progression free survival (PFS) and OS in treatment-naïve metastatic patients (64). Blood molecular analyses are not currently used in daily practice for the management of PDAC despite the very high proportion of *KRAS* mutated tumors. Other studies are needed to confirm these results but circulating exosomal DNA analysis may be considered as a potential screening, diagnosis, or prognosis tool for PDAC management. Castillo et al. performed exosomal proteomic analysis on the “surfaceome” on different human PDAC cell lines, which revealed protein specific expression pattern on exosomal surface (i.e., CLDN4, EpCAM, CD151, LGALS3BP, HIST2H2BE, and HIST2H2BF) (65). This protein panel could be used as a capture assay to enrich pancreatic cancer-specific exosomal cargo, which may improve detection of molecular alterations such as *KRAS* mutations.

### Exosomal miRNA in PDAC EVs

Kumar et al. indicated the presence of a wide variety of RNAs including mRNA, miRNA, lncRNA, tRNA, and piRNA in exosomes in serum of healthy subjects, as well as intraductal papillary mucosal neoplasms and PDAC (66). Exosomes from cancer cells or stromal cells like stellate cells, endothelial cells, or immune cells, carrying miRNAs, participate in tumor pathogenesis and progression by modulating microenvironment and cell phenotypes (1). Ali et al. suggested a crosstalk between pancreatic stellate cells/CAF cells and PDAC cells, resulting in a miR-21/miR-221 over-expression which contributes to aggressiveness to PDAC (67). Takikawa et al. demonstrated that pancreatic stellate cell-derived exosomes contained a variety of miRNAs including miR-21-5p, some of them such as miR-451a were enriched in exosomes compared to stellate cell origin (68). These pancreatic stellate cell-derived exosomes stimulated PDAC cell proliferation, migration, and chemokine (C—X—C motif) ligands 1 and 2 mRNA expression. Yin et al. investigated the role of the exosome-derived tumor-associated macrophage miR-501-3p in the progression of PDAC (69). M2 macrophage-derived exosomal miR-501-3p inhibits tumor suppressor *TGFBR3* gene and facilitates the development of PDAC by activating the TGF-β signaling pathway. Exosomal miRNA involvement in PDAC could provide novel targets for the prevention of tumor progression and/or for the treatment of PDAC.

Detection of exosomal miRNAs in biofluids like serum, plasma, or saliva, being sensitive, non-invasive, and easy to obtain, has a great potential to become a novel screening method for PDAC patients. Despite a lack of standardization in exosomal isolation and measurement, exosomal miR expression seems to be a promising circulating biomarker in PDAC diagnosis or prognosis (70). Lai et al. compared exosomal GPC1 levels to a miR signature for the diagnosis of PDAC. Interestingly, exosomal miR signature (miR-10b, miR-17-5p, miR-21, miR-30c, miR-181a, and let7a) could differentiate PDAC from normal tissue whereas GPC1 did not (70–72). Numerous studies compared the expression of miR in PDAC vs. control group and concluded that miR were highly enriched in pancreatic cancer exosomes (73). Xu et al. found that miR-196a was enriched in PDAC derived exosomes compared with healthy subjects, whereas miR-1246, miR-3976, miR-4306, and miR-4644 expression were significantly increased compared with control groups (74). Interestingly, these miR were also elevated in exosome-depleted serum, but at a low level (75). Zhou et al. identified six exosomal miRNA signatures (miR-122-5p, miR-125b-5p, miR-192-5p, miR-193b-3p, miR-221-3p, and miR-27b-3p) in plasma of pancreatic cancer patients vs. healthy patients (76). These miRNAs could modulate several molecular pathways closely related with pancreatic cancer like p53 and TGF-β signaling pathways. Among these exosomal miRNAs, down-regulation of plasma miR-125b-5p concentration might act as an independent biomarker in predicting OS of pancreatic patients. Zou et al. identified a six-miRNA (let-7b-5p, miR-192-5p, miR-19a-3p, miR-19b-3p, miR-223-3p, and miR-25-3p) panel in serum for pancreatic cancer early and non-invasive diagnosis (77). Their analysis shows significant miR-192-5p, miR-19a-3p, and miR-19b-3p over-expression in both pancreatic tissue and serum-derived exosomes samples. In the same way, exosomal miR-21, miR-191, and miR-451a were also enriched in pancreatic cancer derived exosomes vs. controls. In addition, a high exosomal miR-21 expression was associated with poor OS in pancreatic cancer patients (median OS of 344 vs. 846 days for low expression) (78). These results are consistent with Karasek et al. results which concluded that plasma levels of miR-21 were significantly higher in PDAC compared to healthy controls and associated with poor OS in PDAC (79).

To date, no exosomal biomarkers are used in clinical practice. Exosome isolation from liquid biopsy varies between studies. Further large prospective studies are needed to clarify the potential use of exosomal biomarkers in cancer diagnosis and prognosis.

## EV Imaging *in vivo* and Potential Application in PDAC Metastasis Study

PDAC diagnosis mainly relies on medical imaging and pathological confirmation on tissue sample analysis. EV tracking *in vivo* using multimodal imaging should provide crucial informations in PDAC development. In a context of translational research, a multitude of non-invasive imaging modalities is available both in a preclinical and clinical setting. Multimodal imaging is useful in cancer disease to follow tumor growth, anatomical imaging process, and composition of tumor by contrast or metabolism (80). Non-invasive imaging modalities may provide better understanding of the *in vivo* kinetics of EV release and dissemination during cancer progression. Molecular imaging implies two main methods: direct and indirect EV labeling. During the last decade, the *in vivo* multimodal imaging of small animals, which includes a multitude of techniques, was used in preclinical research. Imaging technology is essential to understand the biodistribution of EVs and their therapeutic implication in pathologies, at the level of intercellular communication between EVs from donor vesicles to receptor cells or distant organs. Thus, it is possible to understand how EVs derived from tumor and/or stromal cells could affect their environment. Currently, multimodal imaging relies on optical imaging, nuclear imaging or Positron Emission Tomography (PET), and Magnetic Resonance Imaging (MRI) (81).

### Optical Imaging for PDAC EV Tracking Analysis

Optical imaging tools and particularly bioluminescence imaging is an indirect cell labeling technique using reporter genes. It is a useful imaging modality considering the easy translation from *in vitro* to *in vivo*. Bioluminescence signal is generated by conversion of chemical energy into visible light due to luciferase enzymes and their substrates in living animals such as *Gaussia* luciferase (GLuc), *Renilla* luciferase (RLuc), *Firefly* luciferase (FLuc), or bacterial luciferase (82–86). In living animals, spatio-temporally real-time non-invasive biological process is a useful for the evaluation of biodistribution, survival, and proliferation of administered cells (87). During last decade, a new optical imaging reporter was used to demonstrate the specific targeting after systemic injection of EVs into the original tumor sites in a thyroid cancer model and for loading anticancer drugs into mesenchymal stem cell-derived exosome mimetics for cancer therapy using *Renilla* Luciferase (Rluc) and *Firefly* Luciferase (Fluc) (88, 89). However, this technique needs to take into consideration the toxicity and half-life of the substrates (e.g., coelenterazine), the poor spatial resolution, limitation of penetration depth, and low quantification accuracy in tissues *in vivo*. Fluorescence imaging is a direct labeling used to observe the initial spatio-temporal biodistribution, localization, and migration of administrated cells. Nonetheless, the using for long-term monitoring cannot be used due to signal dilution by mitotic division of labeled cells and enduring signals from labeled cells (90). However, it is conceivable to image exogenous EV expressing recombinant labeled cell surface biomarkers within the body after a systemic injection and also allows the visualization of intercellular communication through microscopic analysis. Fluorescence measure is achievable thanks to recombinant protein labeling with GFP or RFP. CD63 is commonly used as a marker of exosome (91). Organic fluorescent dyes are also used in direct imaging technique; they are easy to use, their fluorescent signal is stable over time and do not imply genetic engineering of the cells (92, 93). The most commonly used dyes to label cell membrane for imaging are DiR, cy7, DiD, DiL, and PKL. However, labeling with lipophilic dyes promotes clumping of EV. It is important to avoid artifacts by performing many washes prior to injection and/or incubation. EV organotropism could be influenced by EV labeling with lipophilic dye. Optical imaging and its weaknesses make visualization of inner organs impossible and exclude clinical translation.

### Nuclear Imaging for PDAC EV Tracking Analysis

Nuclear imaging or PET allows to visualize *in vivo* three-dimensional measurement of metabolic and molecular processes with a high sensitivity for preclinical and clinical imaging. For cancer imaging the most commonly used tracer is 2-[^18^F] fluoro-2-deoxy-D-glucose ([^18^F]-FDG) (94). In fact, it is possible to visualize the accumulation of radioactive tracers associated with EVs in organs after *in vivo* administration. Combined with other advanced imaging techniques such as MRI or single photon emission computed tomography (SPECT), it is possible to track EVs in tissues and overcome the limitation of tissue penetration of optical imaging. In the study of EVs, this technique uses direct labeling methods and radionuclides like ^111^In-oxine or ^99m^Tc-hexamethylpropyleneamineoxin (HMPAO) (95–97). Nuclear imaging allows much greater penetration in tissues than optical imaging. However, this technique is very expensive and depends on the regulatory policies of radioactive molecules.

### MRI for PDAC EV Tracking Analysis

MRI is a preclinical imaging technique using anatomical modalities which provides high anatomical resolution images with an excellent two- and three-dimensional spatial resolution and no depth limitation. MRI gives an excellent tissue contrast using specialized and personalized protocol like *T*_*2*_-weighted imaged and diffusivity of water molecules with Apparent Diffusion Coefficient (98, 99). The combination of MRI and PET imaging modalities into a single scanner correlates to anatomical findings, morphological information, molecular aspects, and metabolic alterations of cancer diseases (100).

Ultra-small super magnetic particles such as iron oxide or ultra-small super-paramagnetic iron oxide (USPIO) are tracers that allow *T*_2_-weighted to study the EV location in the body (61, 101, 102). These USPIOs can be incorporated by electroporation into EVs or by directly adding them directly into the cell culture medium *in vitro* (102). This technique may be complementary to bioluminescence optical imaging, giving robust, and reliable data. However, before injection, it requires a large amount of labeled EVs to quantify MRI scans. Nonetheless, despite a high-resolution images, the sensitivity with EV-USPIO is lower compared to optical imaging and nuclear imaging (103).

Recent studies have shown the bio-pathological role of EVs in tumor progression, disease diagnosis, and drug-delivery for therapeutic purposes (93, 104). Each imaging modality presents advantages and disadvantages but considering all these different techniques is essential to select the most appropriate one (61). Improving multimodal imaging techniques may benefit to EV future applications including diagnostic and therapeutic approaches in various diseases (38).

### Application in PDAC Metastatic Studies?

PDAC is highly metastatic with poor prognosis, mainly due to delayed detection. Intercellular communication is critical for metastatic progression. PDAC-derived exosomes induce liver pre-metastatic niche formation in naïve mice and consequently increase liver metastatic burden (15). EV tracking *in vivo* using multimodal imaging should provide crucial informations in PDAC development. EVs may also be used as vehicles for drug-delivery and *in vivo* imaging will allow to study their biodistribution (105). A standard operating procedure was established by Mendt et al. to generate engineered exosomes with the ability to target oncogenic KRAS (iExosomes). The clinical-grade iExosomes were tested in multiple *in vitro* and *in vivo* studies to confirm suppression of oncogenic KRAS and an increase in the survival of several mouse models with pancreatic cancer (106, 107).

## Conclusion

EVs present different origins and therefore different contents. By their differences, their contribution in the cross-talk between many cell types is not anecdotal. EV domain is of growing interest. However, EV studies must overcome some difficulties such as the homogenization of the standardization of methodological approaches in EV isolation and a strict respect in the EV characterization. *In vivo* studies by different approaches will contribute to better understand EV impacts in physio-pathological conditions and principally in cancers such as PDAC. EVs may also contribute in patient diagnosis or prognosis. Strategies to promote the therapeutic application of EVs in future clinical studies are more and more considered. EVs of defined cell types could serve as innovative tools for different therapeutic approaches, including anti-tumor therapy, immune-modulatory, and regenerative therapies and drug delivery.

## Author Contributions

BB, SB-P, LN, and J-BO: conception, design, and manuscript writing. All authors: revision and final approval of the manuscript.

## Conflict of Interest

The authors declare that the research was conducted in the absence of any commercial or financial relationships that could be construed as a potential conflict of interest. The handling editor declared a past co-authorship with one of the authors SB-P.
